# Multi-Omics Profiling Identifies Candidate Genes Controlling Seed Size in Peanut

**DOI:** 10.3390/plants11233276

**Published:** 2022-11-28

**Authors:** Yang Liu, Congyang Yi, Qian Liu, Chunhui Wang, Wenpeng Wang, Fangpu Han, Xiaojun Hu

**Affiliations:** 1 Laboratory of Plant Chromosome Biology and Genomic Breeding, School of Life Sciences, Linyi University, Linyi 276000, China; 2 Institute of Genetics and Developmental Biology, Chinese Academy of Sciences, Beijing 100101, China

**Keywords:** peanut, seed size, SNPs and indels, transcriptome, whole genome sequencing

## Abstract

Seed size is the major yield component and a key target trait that is selected during peanut breeding. However, the mechanisms that regulate peanut seed size are unknown. Two peanut mutants with bigger seed size were isolated in this study by ^60^Co treatment of a common peanut landrace, Huayu 22, and were designated as the “big seed” mutant lines (*hybs*). The length and weight of the seed in *hybs* were about 118% and 170% of those in wild-type (WT), respectively. We adopted a multi-omics approach to identify the genomic locus underlying the *hybs* mutants. We performed whole genome sequencing (WGS) of WT and *hybs* mutants and identified thousands of large-effect variants (SNPs and indels) that occurred in about four hundred genes in *hybs* mutants. Seeds from both WT and *hybs* lines were sampled 20 days after flowering (DAF) and were used for RNA-Seq analysis; the results revealed about one thousand highly differentially expressed genes (DEGs) in *hybs* compared to WT. Using a method that combined large-effect variants with DEGs, we identified 45 potential candidate genes that shared gene product mutations and expression level changes in *hybs* compared to WT. Among the genes, two candidate genes encoding cytochrome P450 superfamily protein and NAC transcription factors may be associated with the increased seed size in *hybs*. The present findings provide new information on the identification and functional research into candidate genes responsible for the seed size phenotype in peanut.

## 1. Introduction

Cultivated peanut (*Arachis hypogaea* L.), also known as groundnut, is a major cash crop providing high levels of protein, oil, and other nutrients for humans worldwide. Cultivated peanut is an autogamous allotetraploid legume (AABB, 2n = 40) with homoeologous A and B genomes that are derived from two diploids, *A. duranensis* (AA, 2n = 20) and *A. ipaensis* (BB, 2n = 20) [1,2]. It is widely cultivated in more than 100 countries due to its key role in human nutrition, especially in Asia and Africa, in which the production of peanut accounted for approximately 90% of the global annual production [3,4]. The annual global peanut production has increased rapidly in recent years resulting in a 10 Mt elevation in yield from 2007 (37.51 Mt) to 2017 (47.10 Mt) (http://faostat.fao.org/ ( accessed on 13 June 2022)). However, the rapid increase of world population requires significant increases in crop production, thus there is great potential to enhance peanut global production through increasing the plant productivity.

Plant height, total branch number, and pod and seed/kernel traits have been shown to be closely related to peanut yield [5]. Among these, the pod size/weight and seed size/weight directly influence final peanut production. Several signaling pathways have been shown to control seed size by regulating the growth of maternal tissues, including the ubiquitin–proteasome pathway, G-protein signaling, mitogen-activated protein kinase (MAPK) signaling, phytohormone perception and homeostasis, and some transcriptional regulators [6]. Many genes involved in seed size have been identified in several plants, such as *Hyp O-galactosyltransferase* (*HPGT1*) in *Arabidopsis* [7], *Grain width 2* (*GW2*) in rice [8], and *KERNEL NUMBER PER ROW 6* (*KNR6*) in maize [9]. In the past decade, a number of quantitative trait loci for yield-related traits have been detected on all 20 peanut chromosomes [10,11,12,13], of which 250 QTLs are associated with the seed and/or pod size/weight phenotype. However, due to whole genome duplication events, peanut has a complex genome structure [14,15,16,17]. Thus, the isolation of key genes of yield-related QTLs for seed size/weight variations can be a challenge.

Due to decreased cost and high efficiency, high-throughput RNA sequencing (RNA-Seq) has been extensively used to investigate grain development in various crop species, including peanut [13,18,19,20]. For example, Chen and colleagues explored the developmental dynamics of the peanut pod transcriptome at 11 successive stages and found that the majority of transcripts were differentially expressed along the developmental gradient [18]. In another study, transcriptome analyses were conducted from developing the seeds of two cultivated peanut accessions (Lines 8106 and 8107) and wild *Arachis monticola*, and 4523 genes were identified as specifically expressed during seed development [13]. Recently, Sinha and colleagues reported the *Arachis hypogaea* gene expression atlas for the world’s widest cultivated subsp. Fastigiate, shedding light onto complex regulatory networks in groundnut [20].

There are a limited number of studies that provide information on the regulation of peanut seed size. Considering that peanuts possess the unique growth characteristics of aerial flowers and subterranean fruit, the genes responsible for seed development are likely distinct from those in model plants such as *Arabidopsis* and maize. In the present study, we identified two mutants with a bigger seed size from Huayu 22 (*hybs*) using ^60^Co γ-radiation mutagenesis, and performed morphological, karyotypic, whole genome sequencing, and transcriptome analyses in WT and *hybs* at the key seed developmental stage. The comparative analyses revealed significant differences in the seed size and gene expression between WT and *hybs*, and the combined WGS and RNA-Seq identified two candidate genes that may be responsible for determining peanut seed size. Our work provides useful information for elucidating the complex regulatory mechanism of seed size in peanuts.

## 2. Results

### 2.1. Phenotypic Variation in Seed Size between Hybs and WT Lines

**Figure 1:**
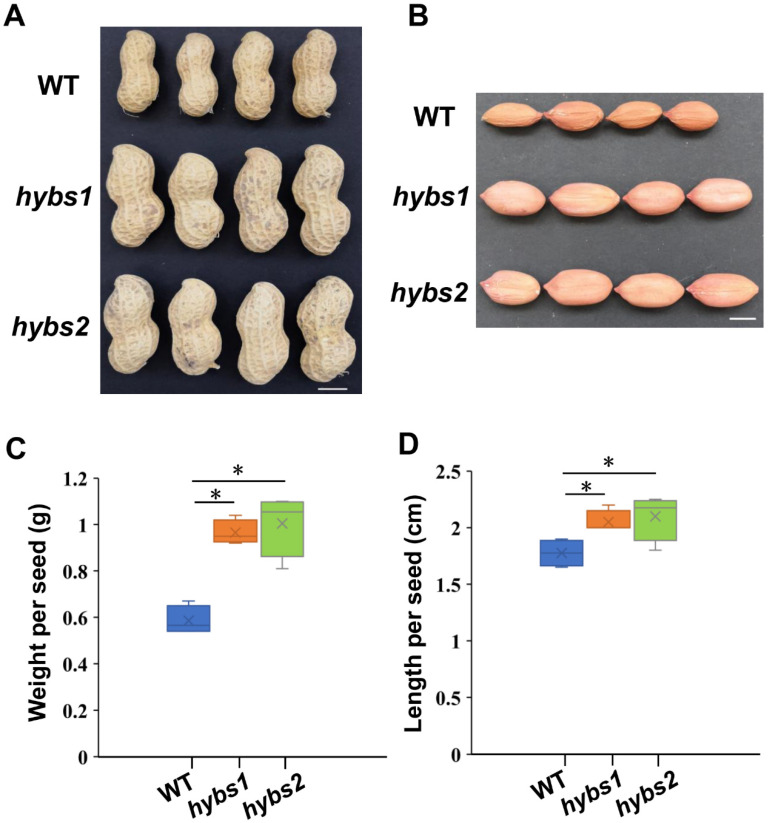
Isolation and characterization of *hybs* mutants. (**A**) Peanut pod phenotypes in *hybs1*, *hybs2*, and WT, Scale bars are 1 cm. (**B**) Peanut seed phenotypes in *hybs1*, *hybs2*, and WT, Scale bars are 1 cm. (**C**) Seed weight of matured seed of *hybs1*, *hybs2*, and WT. Statistically significant differences were analyzed according to four biological replicates (Mann–Whitney *U*-test; * *p* < 0.05). (**D**) Seed length of matured seed of *hybs1*, *hybs2*, and WT. Statistically significant differences were analyzed according to four biological replicates (Mann–Whitney *U*-test; * *p* < 0.05).

In a screen of the ^60^Co-induced mutant collection of *A. hypogaea* L. [cultivar (cv.) Huayu 22], two unique mutants with bigger seeds were isolated and named Huayu 22 big seed1 (*hybs1*) and *hybs2* (Figure 1A). In order to investigate phenotypic differences between *hybs1*, *hybs2*, and WT, we performed a detailed comparation in dry weight and length of seed. We found that the lengths of *hybs1* and *hybs2* seeds were significantly longer (*p* < 0.01) than those of WT after harvest (Figure 1B,D). In agreement with the observed increase in seed length, the dry weight of the seed increased from 0.58 g in WT to 0.97 g and 1.01 g in *hybs1* and *hybs2*, respectively (Figure 1C,D). Taken together, these results suggested that we successfully isolated and characterized two mutants of peanuts, which exhibit a bigger seed size phenotype.

### 2.2. Hybs Did Not Show Large Chromosome Structural Variations by Karyotype Analysis and Whole-Genome Resequencing

**Figure 2:**
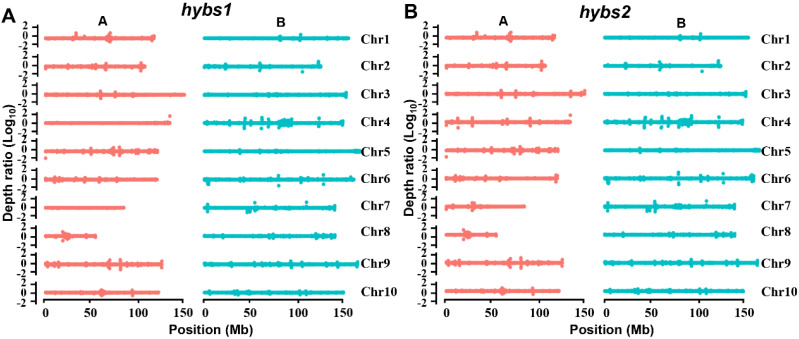
Depth−of−coverage of sequence reads from WT, *hybs1*, and *hybs2* along the chromosomes. (**A**,**B**) Distribution of the sliding window average of depth ratio in *hybs1* (**A**) and *hybs2* (**B**). The window size was 100 kb. The depth ratio was calculated by dividing the depth−of−coverage of mutant by the depth−of−coverage of WT; the ratio was then log_10_ transformed. Red: A subgenome; Skyblue: B subgenome.

To investigate whether *hybs1* and *hybs2* displayed extensive changes in chromosome structures, we performed WGS of the WT, as well as the *hybs1* and *hybs2* mutants (on 8× coverage each plant) (Supplementary Material Table S1). In total, 253 to 302 million reads were obtained. After removing short reads with poor quality and polymerase chain reaction (PCR) duplicates, 130 to 159 million reads with an average of 98.9% mapping ratio for the three lines were obtained that aligned to the Tifrunner reference genome [17]. The average depth-of-coverage was 8.94 for WT, 7.23 for *hybs1*, and 8.60 for *hybs2*. If there was a large deletion on the chromosome of mutants, a ratio decrease in depth-of-coverage in the chromosome should be uniquely observed. To identify genomic regions where depth-of-coverage uniquely decreased in the mutants, we analyzed the moving average of depth-of-coverage per 100 kb over the chromosomes. The ratio was calculated by dividing the depth-of-coverage of mutants by the depth-of-coverage of WT; the ratio was then log_10_ transformed. We found that the ratio was relatively stable at 0 along the whole chromosome (Figure 2A,B), indicating no large segment deletions (more than 1 Mb), duplications, or translocations in the mutants. In order to validate the WGS observations, we designed two cocktails, Multiplex #3 and Multiplex #4 [21]. Multiplex #3 included FAM-modified oligo TIF-439, oligo TIF-185-1, oligo TIF-134-3, and oligo TIF-165. Multiplex #4 included TAMRA-modified oligo Ipa-1162, oligo Ipa-1137, oligo DP-1, and oligo DP-5. Each cocktail enabled the establishment of a genome map-based karyotype after sequential fluorescence in situ hybridization (FISH)/genomic in situ hybridization (GISH). The results showed no evidence for large structural abnormalities of chromosomes (Figure S1). Taken together, these results suggested that the large chromosome structural variations are likely not the causes of the mutant phenotypes.

### 2.3. Single Nucleotide Polymorphism (SNP) and Indel Analysis in WT and hybs Mutants

Though we did not find large chromosome structural variations, we suspected SNPs or indels were potentially induced by ^60^Co. To comprehensively evaluate ^60^Co-induced effects, we analyzed our WGS data of the WT, as well as the *hybs1* and the *hybs2* plants for the identification of SNPs and indels. We performed SNP/indel calling separately for the three samples. As shown in Table 1, compared to the Tifrunner reference genome, 370,912 SNPs and 98,445 indels were present as variants in the WT plant, as a result of genetic variations between Huayu 22 and Tifrunner reference genome. In addition, compared to the Tifrunner reference genome, we identified 334,753 SNPs and 84,850 indels, and 368,220 SNPs and 97,956 indels in the *hybs1* and *hybs2* plants, respectively. We also identified 94,719 SNPs and 32,012 indels in the *hybs1*, and 111,194 SNPs and 38,600 indels in the *hybs2* that were not present in the WT plant (Table 1). For this reason, they were named “^60^Co-induced SNPs/indels”. To evaluate the genomic distribution of ^60^Co-induced SNPs/indels, we calculated the number of ^60^Co-induced SNPs/indels in every 5 Mb window and observed that the mutants were almost identical in terms of SNPs/indels distribution pattern (Figure S2). The ^60^Co-induced SNP/indel densities for the mutants were unevenly distributed over the chromosomes with a chromosome-level trend increasing toward pericentromeres and centromeres (Figure S2), and there are more variations in subgenomes B (Figure S3).

To further dissect the distribution of these ^60^Co-induced SNPs/indels, we annotated the genomic distribution of SNPs/indels by dividing the genome into six classes that included five classes of genic regions (promoter, 5′ UTR, 3′ UTR, coding exon, and intron) and intergenic regions. We calculated the expected numbers of ^60^Co-induced SNPs/indels in these five categories by randomly selecting an equal number of control sites in the genome and comparing them with the ^60^Co-induced SNPs/indels of *hybs1* and *hybs2* (observed number). Though the majority of the genomic variants occurred in the intergenic region (Table 2), the ratios of observed number/expected number of indels were much higher in 5′ UTR (1.57 and 1.66), 3′ UTR (1.02 and 1.03), and intron regions (1.24 and 1.23) than in exon (0.57 and 0.58), intergenic (0.86 and 0.86), promoter (0.45 and 0.30), and terminator regions (0.43 and 0.44) in *hybs1* and *hybs2*, respectively, suggesting that ^60^Co-induced indels were preferentially enriched in 5′ UTR, 3′ UTR and intron regions (Figure S4A). However, we did not detect ^60^Co-induced SNP enrichment in all six genomic elements (Figure S4B).

**Figure 3:**
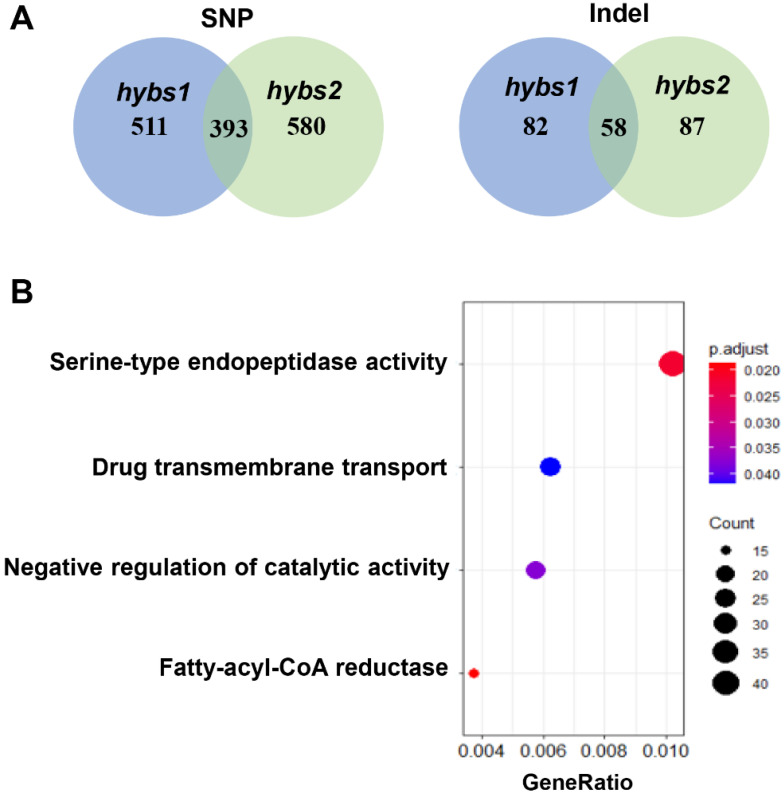
Annotation of SNPs and indels. (**A**) Number of genes that large-effect SNPs and indels occurred. (**B**) GO enrichment analysis of genes with large-effect SNPs and indels.

Since large-effect genetic variants cause non-functional proteins leading to various phenotypic changes, we were prompted to investigate ^60^Co-induced SNPs and indels with large-effect in *hybs1* and *hybs2*. The large-effect variants include the disruption of splicing sites, start codon losses, stop codon losses, and stop codon gains. For all the comparisons, large-effect SNPs largely resulted from the splice site donor (843 for *hybs1* and 1012 for *hybs2*) (Table 2). In contrast, the numbers of large-effect indels were relatively small even among different origin of variants (Table 2). We found that large-effect SNPs with these variations occurred in 511 genes in *hybs1*, and 580 genes in *hybs2*, among which 393 were shared by *hybs1* and *hybs2*. In the case of large-effect indels, indels with these variations occurred in 82 genes in *hybs1*, and 87 genes in *hybs2*, among which 58 were shared by *hybs1* and *hybs2* (Figure 3A). In order to investigate the putative functions of these shared genes (SNPs/indels), gene ontology (GO) biological process enrichment analysis was performed. The result indicated multiple functions of these 451 genes, involved in pathways ranging from the serine-type endopeptidase activity and negative regulation of catalytic activity to the fatty-acyl-CoA reductase (Figure 3B).

### 2.4. RNA-Seq Analyses on Seed at the Developmental Stage of WT, Hybs1, and Hybs2

**Figure 4:**
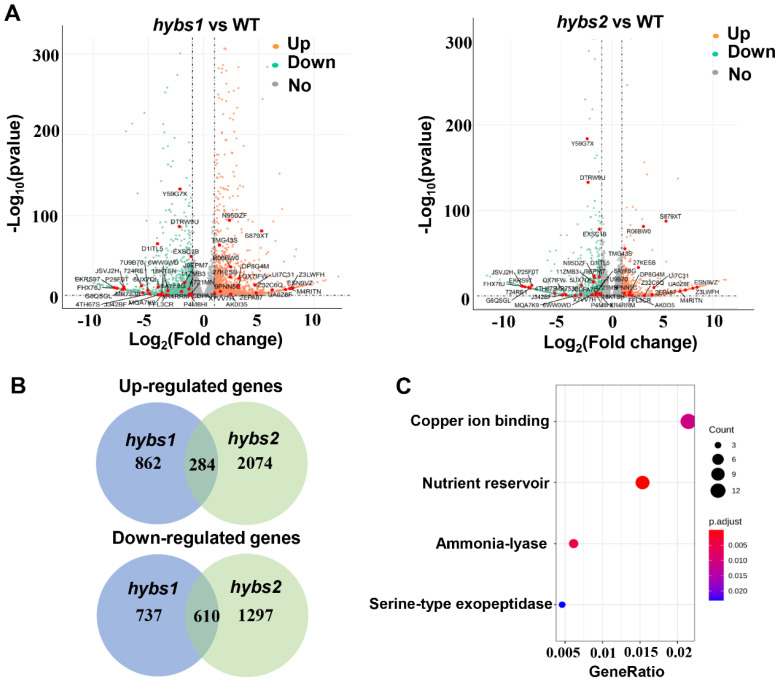
Differentially expressed genes in *hybs* mutants compared to WT. (**A**) The red dots indicate genes with significant up−regulation, and the cyan dots indicate genes with significant down−regulation. The 45 potential candidate genes were marked on the volcano plot. (**B**) The number of up−regulated and down−regulated genes shared by *hybs1* and *hybs2*. (**C**) The enriched biological processes for shared DEGs in the GO enrichment analysis.

In order to check whether these genes showed a transcriptional change as a result of SNP/indel mutations, we performed RNA-Seq analyses on seed between WT and *hybs* mutants at 20 DAF with three independent biological replicates. A total of nine samples were sequenced, and 15.02–17.86 million high-quality reads were obtained for each sample (Supplementary Material Table S2). Approximately 88.70–91.04% of the high-quality reads were mapped to the peanut tetraploid genome, Tifrunner.gnm1.KYV3. Genes with FPKM <0.1 in all samples were removed, and 38,846, 39,017, and 37,425 genes were detected in WT, *hybs1*, and *hybs2*, respectively. The number of genes with very high (>50), high (10 ≤ FPKM < 50), moderate (2 ≤ FPKM < 10), and low (0.1 ≤ FPKM < 2) expression levels accounted for approximately 2%, 12%, 41%, and 45%, respectively, in different samples (Figure S5A). To identify differentially expressed genes (DEGs), a stringent value of the false discovery rate (FDR) < 0.05 and the absolute fold change (|FC|) ≥ 2 were used as thresholds; results showed that a total of 2493 and 4265 DEGs were detected in *hybs1* and *hybs2* seed compared with WT seed (Figure 4A), with 1146 and 1347 up-/down-regulated genes for *hybs1* and 2358 and 1907 up-/down-regulated genes for *hybs2*, respectively (Figure S5B,C). We found 284 up-regulated genes and 610 down-regulated genes shared by *hybs1* and *hybs2* (Figure 4B). The up-/down-regulated genes shared by the DEG sets may contribute to increased seed size during seed development, so we performed a GO enrichment analysis. The results revealed that most DEGs were correlated with four major biological processes, including copper ion binding, nutrient reservoir activity, ammonia-lyase activity, and serine-type exopeptidase activity (Figure 4C).

**Figure 5:**
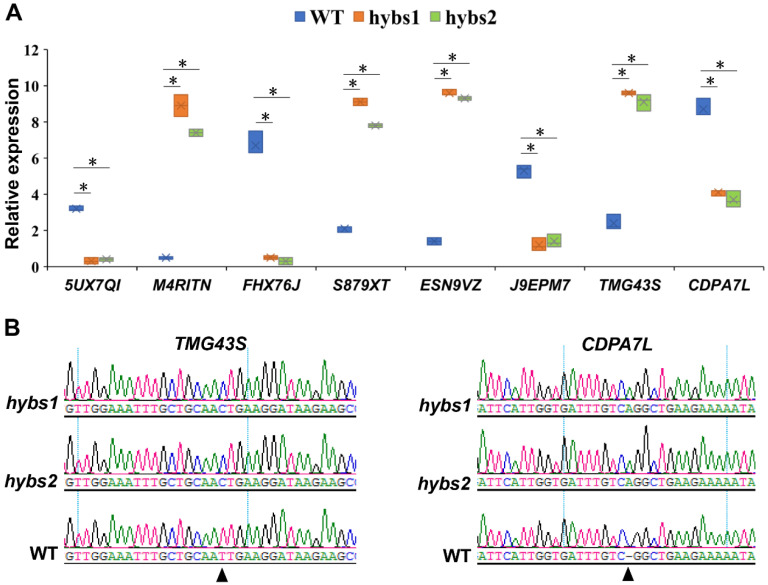
qRT-PCR verification of DEGs between *hybs* and WT lines. (**A**) qRT-PCR analysis of 8 genes expression in seed samples in WT and *hybs* plants. (Mann-Whitney *U*-test; * *p* < 0.01). (**B**) Results of Sanger sequencing for the *hybs* mutants and WT. The arrows indicated the mutations.

We next combined the DEGs with the results of the large-effect SNPs/indels analysis. Only genes both differentially expressed between *hybs* mutants and WT and containing large-effect SNPs/indels were considered to be candidate genes for regulating peanut seed size. This analysis led to the identification of 45 potential candidate genes. We applied quantitative reverse-transcription PCR (qRT-PCR) to eight genes randomly selected from the 45 potential candidate genes to evaluate the accuracy of the RNA-Seq data. The results revealed that the overall expression trends of these eight genes were consistent with those obtained from RNA-seq analysis (Figure 4A and Figure 5A). Within the most common genes, two putative candidate genes, *Tifrunner.gnm1.ann1.TMG43S* and *Tifrunner.gnm1.ann1.CDPA7L* were selected, which were significantly up-regulated and down-regulated, respectively, in the *hybs1* and *hybs2* mutant (Figure 5A). Using PCR amplification and DNA sequencing, we validated the SNP and indel variations in the two candidate genes from *hybs* mutants (Figure 5B, Table 3). Specifically, the gene *Tifrunner.gnm1.ann1.TMG43S* encodes a Cytochrome P450 superfamily protein, which has been suggested to be positively correlated with wheat seed size [22]. Another gene contains a conserved NAC domain (Table 3). The NAC transcription factors are implicated in seed storage protein synthesis, secondary cell wall biosynthesis, xylem vessel element formation and leaf senescence [23]. Recently, three NAC TFs have been predicted to be associated with the seed size of rice [24]. However, whether or not these genes are responsible for the bigger seed phenotype in peanut will need to be confirmed by further functional studies.

## 3. Discussion

Peanut is one of the most important sources of oilseed. Thus, as the demand for oil is ever-increasing, there is an urgent need to breed new peanut varieties with high yields, a characteristic that is dependent on seed size. Several major quantitative trait loci (QTLs) related to peanut pod size have been obtained in recent years [12,25,26,27,28,29,30,31]. Luo and colleagues found three major consistent and stable QTLs for pod size and weight, which were co-localized in a 3.7 cm interval on chromosome A05 [27]. Furthermore, Luo and colleagues identified three and two major QTLs controlling pod weight and size on chromosomes A07 and A05, respectively, in an RIL population across four environments [28]. Recently, Chu and colleagues identified nine QTL, in which the locus on linkage groups (LGs) A05 explained up to 66% of the phenotypic variation for all measured pod and seed traits. Alyr and colleagues identified one QTL associated with pod and seed size in a 168.37 kb interval on chromosome A07 [29]. In addition to these QTLs associated with seed traits, Ma and colleagues investigated comprehensive lncRNA profiles derived from the seed development in two peanut recombinant inbred lines (RIL8) that differ in seed size. They provided new information on lncRNA-mediated regulatory roles in peanut seed development. Though great progress has been achieved in the last decade, understanding of the molecular mechanisms that underlie seed size remains limited. In this study, we combined WGS and RNA-seq to rapidly characterize two seed mutants *hybs* that showed a bigger seed size. In a previous study, ^60^Co treatment led to two large deletions on the wheat chromosome, which is responsible for the reduced spike and grain lengths [32]. Similarity, genome-wide comparisons of depth-of-coverage between WT and gamma-irradiated mutants of wheat “30579” detected ~130 Mb deletion on the short arm of chromosome 5D in the mutant genome [33]. However, our karyotype analysis and WGS did not identify large chromosome structural variations, while identifying large-effect variants (SNPs and indels) that led to gene product change induced by ^60^Co, which may be involved in the regulation of seed size. Assisted with gene expression data, we found that there are nearly more than twice as many DEGs in *hybs2* (4265) than in *hybs1* (2493). We performed three biological replicates for each case and a stringent value of the false discovery rate (FDR) < 0.05 and the absolute fold change (|FC|) ≥ 2 were used as thresholds. Therefore, it is not likely a consequence of data processing. Considering the randomness of radiation in genetic mutations, it may exert different influences on gene expression, which resulted in the different number of DEGs. In addition to the phenotypic variation in seed size between *hybs* and WT lines, we also observed that the plant height was different between *hybs1* and *hybs2* (Figure S6). *Hybs1* was lower than WT, while *hybs2* was taller than WT, indicating that ^60^Co induced unique mutations leading more specific genes expression pattern between *hybs1* and *hybs2*. Combining the DEGs with the results of the large-effect SNPs/indels analysis, we found 45 genes showing gene product and expression level change. Two candidate genes and their homologous genes in wheat and rice have been shown to participate in seed size regulation. Our multi-omics approach provides a strategy to rapidly detect potential loci and genes in peanut.

Among the two genes, one is an NAC transcription factor. The NAC TFs control plant development, senescence, morphogenesis, and abiotic stress tolerances [34]. NAC TFs also participate in the regulation of grain yield, seed size and biomass [35]. For example, the over-expression of root specific *OsNAC5* in rice plants showed an increment in grain yield of 9–23% under normal conditions [36]. In another case, three NAC TF encoding genes, namely *ONAC020*, *ONAC026*, and *ONAC023* exhibited significantly strong association with seed size in rice [37]. In contrast to the positive up-regulation of yield by NAC members, a recent study showed overexpression of miR164b or down-regulation of *OsNAC2* led to decreased panicle length and grain yield in the main panicle [38]. In our study, we found that ^60^Co treatment induced a base change from “C” to “CA”, which led to the frameshift mutation in both *hybs1* and *hybs2*. At the same time, the expression level of the NAC showed a significant decrease in mutants. As NAC proteins are represented by a conserved N-terminal DNA binding domain and a variable transcription regulatory region at the C-terminus, which plays a role in either transcriptional activation or repression of genes [35], we suspected the identified NAC in peanut may be involved in repression of seed size. Another gene encodes a cytochrome P450 protein. The cytochrome P450 (CYP) family is one of the largest families of plant proteins [39,40], which have been shown to affect the process of seed development [41,42]. For example, the overexpression of wheat *CYP78A3* induced the production of more cells in the seed coat, leading to an 11–48% increase in Arabidopsis seed size [22]. *SlKLUH*, an orthologous gene of *CYP78A5* in tomato (*Solanum lycopersicum*), has also been proved to regulate fruit mass by shortening the cell division period [43,44,45]. In our study, we found that the CYP gene has a base change, which led to the amino acid substitution in both *hybs1* and *hybs2*. Interestingly, the expression level of *CYP* was significantly increased, which may be responsible for the bigger seed size phenotype. Further research is required, in particular on the functional verification of these genes in the regulation of seed size in peanut.

## 4. Materials and Methods

### 4.1. Plant Materials

The Chinese elite cultivar Huayu 22 is high yielding, high quality and resistant to several diseases and has wide adaptation. To generate mutants, the dry seeds of “Huayu 22” were irradiated with gamma-rays from Co-60 (1000 Gy). The irradiated seeds were sown to obtain the M1. Two mutants with big seed size were selected in M2 to evaluate the seed traits, including the seed length, width, and weight according to a previous study [46]. The mutants and Huayu 22 lines were planted in the experimental fields in Linyi (at N 34.22°, E 118.05°), Shandong Province (planted in May and harvested in September of 2021). The field experiments followed a randomized block design with three replications as described [11]. Each plant was harvested individually at its maturity to prevent loss from over-ripening. A total of 12 representative seeds from 3 individual plants were selected for each biological experiment from both lines.

### 4.2. DNA Extraction and WGS

Genomic DNA was extracted from young leaves of *hybs* mutants and WT plants using a plant genomic DNA extraction kit (Bioteke, Beijing, China) according to the user manual. The RNase A was used to remove RNA contamination. The concentration and quality of each genomic DNA were measured using the Qubit^®^ DNA Assay Kit in Qubit^®^ 2.0 Fluorometer (Life Technologies, Carlsbad, CA, USA) and 1% agarose gel electrophoresis, respectively. Only genomic DNA samples with an OD260/280 value ranging from 1.8 to 2.2 were considered good quality DNA. Approximately 0.5 µg of DNA was collected to construct sequencing libraries. Library construction and sequencing services were provided by Novogene (Beijing, China). Raw reads were quality-trimmed as described [47] to remove adaptor and low-quality bases. The quality-controlled reads were then aligned to the tetraploid peanut reference genome Tifrunner.gnm1. KYV3 [17] (https://peanutbase.org(accessed on 13 June 2022)) using Burrows Wheeler Aligner BWA-MEM [48]. Low mapping quality reads and duplicated reads were removed by samtools (version 1.3.1) and Picard tools (http://broadinstitute.github.io/picard (accessed on 13 June 2022)) (version 2.26.9). The SNPs and indels were identified using GATK (The Genome Analysis Toolkit, version 3.8.1) [49] and bcftools (Tools for variant calling and manipulating VCFs and BCFs, version 1.15). The resulting sequences were filtered using GATK (parameters: -T VariantFiltration—filterExpression “QD < 2.0 || FS > 200.0 || SOR > 10.0 || MQRankSum < −12.5 || ReadPosRankSum < −8.0”—filterName “PASS”) [49]. 

### 4.3. RNA Isolation, RNA-Seq, and Differential Genes Expression Analyses

We planted the mutants and Huayu 22 parallelly in the same experimental fields in Linyi as described in the section on plant materials. The seed samples were collected in 2021 20 days after flowering from nine different plants. Three biological replicates were performed in each case. WT, *hybs1*, and *hybs2* seed samples were then rapidly frozen in liquid nitrogen and stored at −80 °C. Total RNA was extracted from peanut seeds using Trizol reagent (Invitrogen, Carlsbad, CA, USA) as previously reported [50]. RNA integrity and quality were checked by using an Agilent 2100 bioanalyzer (Agilent Technologies, Carpinteria, CA, USA) and Qubit (Invitrogen, Carlsbad, CA, USA). Subsequently, a total of 9 libraries were subjected to RNA sequencing using an Illumina HiSeq2500 platform (Annoroad, Beijing, China). Raw reads were quality-trimmed as described in [47] to remove adaptor and low-quality bases. The quality-controlled reads were then aligned to the tetraploid peanut reference genome Tifrunner.gnm1.KYV3 using HISAT2 [51]. Only the uniquely mapped reads identified across the entire genome were retained for further analyses. Reads counts of each gene were summarized by using HTseq-count [52] with options “-f bam -r name -s no -a 0”. DEseq2 was applied to identify differentially expression genes between samples via algorithms, and the resultant *p* values were adjusted using a Benjamini and Hochberg’s correction to control for FDR [53]. The differentially expressed genes were identified with criteria “FDR < 0.05 and fold change ≥ 2”. For DEGs, the GO enrichment analysis of DRGs was performed by clusterProfiler, which supports the statistical analysis and visualization of functional profiles for genes and gene clusters [54]. The terms/pathways in GO analyses with a q-value (FDR) < 0.05 were considered to be significantly enriched. 

### 4.4. qRT-PCR Analysis

We performed qRT-PCR using a Qiagen SuperScript II Kit (Qiagen, Hilden, Germany), conducted on a 7900 HT Fast Real-Time PCR system (Applied Biosystems, Waltham, MA, USA). Gene expression of both lines (*hybs* and WT) was detected for seed samples at DAF 20, and three repeats of each reaction for individual genes were performed. The relative expression of each gene among different samples was calculated by using the 2^−∆∆Ct^ method with normalization to the internal reference actin gene. Primer Premier 3.0 was used to design gene-specific primers for eight genes and these primer sequences are listed in Supplementary Material Table S3.

### 4.5. Fluorescence in Situ Hybridization (FISH) and Genomic in Situ Hybridization (GISH)

FISH and GISH analyses of different plants were conducted as previously published [55]. To establish the karyotype, two multiplex probe cocktails, named Multiplex #3 and Multiplex #4, were developed. Multiplex #3 included FAM-modified TIF-439, TIF-185-1, TIF-134-3 and TIF-165-3; and Multiplex #4 included TAMRA-modified Ipa-1162, Ipa-1137, DP-1 and DP-5 (Supplementary Material Table S3). The root tip cells were treated with an equal amount of probe. In both cases, chromosomes were counterstained with 4′, 6-diamidino-2-phenylindole. Images were acquired using confocal microscopy (Zeiss Cell Observer SD) and were processed with Adobe Photoshop CS 6.0. The GISH procedure was performed using total genomic DNAs of *A. duranensis* (green) and *A. ipaensis* (red) as the probes.

### 4.6. Statistical Analysis

Statistical analysis of *hybs* mutants and WT lines was carried out via an independent Mann–Whitney *U*-test. The correlation coefficient between gene expression and spike length was calculated using the R function “cor ()” based on Pearson’s method. 

## Figures and Tables

**Table 1:** Number of SNPs and Indels Identified in WT, *hybs1*, and *hybs2*.

Variant	WT vs. Ref	*Hybs1* vs. Ref	*Hybs2* vs. Ref	*Hybs1* Private Variations	*Hybs2* Private Variations
SNP	370,912	334,753	368,220	94,719	111,194
Indel	98,445	84,850	97,956	32,012	38,600

**Table 2:** The number of SNPs/indels in six genomic elements and the number of large-effect SNPs/indels.

	*Hybs1*		*Hybs2*	
	SNP	Indel	SNP	Indel
Promoter	6114	3375	6787	3995
Terminator	5321	2826	5929	3299
5′ UTR	336	298	359	361
3′ UTR	520	308	553	383
Exon	2919	863	3291	1037
Intron	4220	2295	5053	2745
Intergenic	87580	28870	102850	34838
Splice site acceptor	41	48	26	44
Splice site donor	843	27	1012	20
Start lost	8	13	6	16
Stop gained	4	21	4	19
Stop lost	6	3	7	5

**Table 3:** Summary of identified candidate genes related to the seed size phenotype based on RNA-seq and WGS.

Mutant	Gene_ID	Chromosome	Physical Position (bp)	Reference	Variation	SNP/Indel Effect	Predicated Gene Function
*Hybs1*	*Tifrunner.gnm1.ann1.TMG43S*	B04	21,074,785	T	C	Amino acid change	Cytochrome P450 superfamily protein
*Hybs2*	*Tifrunner.gnm1.ann1.TMG43S*	B04	21,074,785	T	C	Amino acid change	Cytochrome P450 superfamily protein
*Hybs1*	*Tifrunner.gnm1.ann1.CDPA7L*	A08	30,827,924	C	CA	Frameshift	NAC transcription factor
*Hybs2*	*Tifrunner.gnm1.ann1.CDPA7L*	A08	30,827,924	C	CA	Frameshift	NAC transcription factor

## Data Availability

The RNA-Seq and WGS data generated in this study have been submitted to the NGDC Genome Sequence Archive (GSA; https://bigd.big.ac.cn/gsa/ (accessed on 17 October 2022)) under accession number CRA008269.

## Supplementary Materials

The following supporting information can be downloaded at: https://www.mdpi.com/article/10.3390/plants11233276/s1, Figure S1. Karyotypic analysis of WT, *hybs1*, and *hybs2*. Figure S2. Manhattan plot for ^60^Co-induced SNPs/indels. Figure S3. The number of 60Co-induced SNPs/indels in *hybs1* and *hybs2*. Figure S4. Relative enrichment of SNPs/Indels in different genomic elements. Figure S5. RNA-Seq analyses of *hybs1* and *hybs2*. Figure S6. Plant height of WT, *hybs1*, and *hybs2*; Table S1. Summary of sequencing data quality of WGS. Table S2. Summary of sequencing data quality of RNA-seq. Table S3. List of oligos and primer sequences used in this study.

## References

[1] Kochert G., Stalker H.T., Gimenes M., Galgaro L., Lopes C.R., Moore K. (1996). RFLP And Cytogenetic Evidence on the Origin and Evolution of Allotetraploid Domesticated Peanut, *Arachis hypogaea* (Leguminosae). Am. J. Bot..

[2] Zhang X., Zhang K., Luo L., Lv Y., Li Y., Zhu S., Luo B., Wan Y., Zhang X., Liu F. (2022). Identification of Peanut Aux/IAA Genes and Functional Prediction during Seed Development and Maturation. Plants.

[3] Abdurakhmonov I.Y., Abdukarimov A. (2008). Application of association mapping to understanding the genetic diversity of plant germplasm resources. Int. J. Plant Genom..

[4] Zhao J., Huang L., Ren X., Pandey M.K., Wu B., Chen Y., Zhou X., Chen W., Xia Y., Li Z. (2017). Genetic Variation and Association Mapping of Seed-Related Traits in Cultivated Peanut (*Arachis hypogaea* L.) Using Single-Locus Simple Sequence Repeat Markers. Front. Plant Sci..

[5] Shirasawa K., Koilkonda P., Aoki K., Hirakawa H., Tabata S., Watanabe M., Hasegawa M., Kiyoshima H., Suzuki S., Kuwata C. (2012). *In silico* Polymorphism Analysis for the Development of Simple Sequence Repeat and Transposon Markers and Construction of Linkage Map in Cultivated Peanut. BMC Plant Biol..

[6] Li N., Xu R., Li Y. (2019). Molecular Networks of Seed Size Control in Plants. Annu. Rev. Plant Biol..

[7] Ogawa-Ohnishi M., Matsubayashi Y. (2015). Identification of Three Potent Hydroxyproline O-galactosyltransferases in Arabidopsis. Plant J..

[8] Yamaguchi K., Yamamoto T., Segami S., Horikawa M., Chaya G., Kitano H., Iwasaki Y., Miura K. (2020). gw2 Mutation Increases Grain Width and Culm Thickness in Rice (*Oryza sativa* L.). Breed. Sci..

[9] Jia H., Li M., Li W., Liu L., Jian Y., Yang Z., Shen X., Ning Q., Du Y., Zhao R. (2020). A Serine/threonine Protein Kinase Encoding gene KERNEL NUMBER PER ROW6 Regulates Maize Grain Yield. Nat. Commun..

[10] Lu Q., Liu H., Hong Y., Li H., Liu H., Li X., Wen S., Zhou G., Li S., Chen X. (2018). Consensus Map Integration and QTL Meta-analysis Narrowed a Locus for Yield Yraits to 0.7 cM and Refined a Region for Late Leaf Spot Resistance Traits to 0.38 cM on Linkage Group A05 in Peanut (*Arachis hypogaea* L.). BMC Genom..

[11] Zhang S., Hu X., Miao H., Chu Y., Cui F., Yang W., Wang C., Shen Y., Xu T., Zhao L. (2019). QTL Identification for Seed Weight and Size Based on a High-density SLAF-seq Genetic Map in Peanut (*Arachis hypogaea* L.). BMC Plant Biol..

[12] Gangurde S.S., Wang H., Yaduru S., Pandey M.K., Fountain J.C., Chu Y., Isleib T., Holbrook C.C., Xavier A., Culbreath A.K. (2020). Nested-association Mapping (NAM)-based Genetic Dissection Uncovers Candidate Genes for Seed and Pod Weights in Peanut (*Arachis hypogaea*). Plant Biotechnol. J..

[13] Li Z., Zhang X., Zhao K., Zhao K., Qu C., Gao G., Gong F., Ma X., Yin D. (2021). Comprehensive Transcriptome Analyses Reveal Candidate Genes for Variation in Seed Size/Weight During Peanut (*Arachis hypogaea* L.) Domestication. Front. Plant Sci..

[14] Bertioli D.J., Cannon S.B., Froenicke L., Huang G., Farmer A.D., Cannon E.K.S., Liu X., Gao D., Clevenger J., Dash S. (2016). The Genome Sequences of *Arachis duranensis* and *Arachis ipaensis*, the Diploid Ancestors of Cultivated Peanut. Nat. Genet..

[15] Yin D., Ji C., Ma X., Li H., Zhang W., Li S., Liu F., Zhao K., Li F., Li K. (2018). Genome of an Allotetraploid Wild Peanut *Arachis monticola*: A de novo assembly. Gigascience.

[16] Zhuang W., Chen H., Yang M., Wang J., Pandey M.K., Zhang C., Chang W.-C., Zhang L., Zhang X., Tang R. (2019). The Genome of Cultivated Peanut Provides Insight into Legume Karyotypes, Polyploid Evolution and Crop Domestication. Nat. Genet..

[17] Bertioli D.J., Jenkins J., Clevenger J., Dudchenko O., Gao D., Seijo G., Leal-Bertioli S.C.M., Ren L., Farmer A.D., Pandey M.K. (2019). The Genome Sequence of Segmental Allotetraploid Peanut *Arachis hypogaea*. Nat. Genet..

[18] Chen X., Yang Q., Li H., Li H., Hong Y., Pan L., Chen N., Zhu F., Chi X., Zhu W. (2016). Transcriptome-wide Sequencing Provides Insights into Geocarpy in Peanut (*Arachis hypogaea* L.). Plant Biotechnol. J..

[19] Clevenger J., Chu Y., Scheffler B., Ozias-Akins P. (2016). A Developmental Transcriptome Map for Allotetraploid *Arachis hypogaea*. Front. Plant Sci..

[20] Sinha P., Bajaj P., Pazhamala L.T., Nayak S.N., Pandey M.K., Chitikineni A., Huai D., Khan A.W., Desai A., Jiang H. (2020). Arachis hypogaea Gene Expression Atlas for Fastigiata Subspecies of Cultivated Groundnut to Accelerate Functional and Translational Genomics applications. Plant Biotechnol. J..

[21] Fu L., Wang Q., Li L., Lang T., Guo J., Wang S., Sun Z., Han S., Huang B., Dong W. (2021). Physical Mapping of Repetitive Oligonucleotides Facilitates the Establishment of a Genome Map-based Karyotype to Identify Chromosomal Variations in Peanut. BMC Plant Biol..

[22] Ma M., Wang Q., Li Z., Cheng H., Li Z., Liu X., Song W., Appels R., Zhao H. (2015). Expression of TaCYP78A3, a Gene Encoding Cytochrome P450 CYP78A3 Protein in Wheat (*Triticum aestivum* L.), Affects Seed Size. Plant J..

[23] Shen L., Luo G., Song Y., Xu J., Ji J., Zhang C., Gregová E., Yang W., Li X., Sun J. (2021). A Novel NAC Family Transcription Factor SPR Suppresses Seed Storage Protein Synthesis in Wheat. Plant Biotechnol. J..

[24] Dwivedi N., Maji S., Waseem M., Thakur P., Kumar V., Parida S.K., Thakur J.K. (2019). The Mediator Subunit OsMED15a is a Transcriptional Co-regulator of Seed Size/weight–modulating Genes in Rice. Biochim. Et Biophys. Acta (BBA)-Gene Regul. Mech..

[25] Huang L., He H., Chen W., Ren X., Chen Y., Zhou X., Xia Y., Wang X., Jiang X., Liao B. (2015). Quantitative Trait Locus Analysis of Agronomic and Quality-related Traits in Cultivated Peanut (*Arachis hypogaea* L.). Theor. Appl. Genet..

[26] Chen W., Jiao Y., Cheng L., Huang L., Liao B., Tang M., Ren X., Zhou X., Chen Y., Jiang H. (2016). Quantitative Trait Locus Analysis for Pod- and Kernel-related Traits in the Cultivated Peanut (*Arachis hypogaea* L.). BMC Genet..

[27] Luo H., Ren X., Li Z., Xu Z., Li X., Huang L., Zhou X., Chen Y., Chen W., Lei Y. (2017). Co-localization of major quantitative trait loci for pod size and weight to a 3.7 cM interval on chromosome A05 in cultivated peanut (*Arachis hypogaea* L.). BMC Genom..

[28] Luo H., Guo J., Ren X., Chen W., Huang L., Zhou X., Chen Y., Liu N., Xiong F., Lei Y. (2018). Chromosomes A07 and A05 associated with stable and major QTLs for pod weight and size in cultivated peanut (*Arachis hypogaea* L.). Theor. Appl. Genet..

[29] Alyr M.H., Pallu J., Sambou A., Nguepjop J.R., Seye M., Tossim H.-A., Djiboune Y.R., Sane D., Rami J.-F., Fonceka D. (2020). Fine-Mapping of a Wild Genomic Region Involved in Pod and Seed Size Reduction on Chromosome A07 in Peanut (*Arachis hypogaea* L.). Genes.

[30] Chu Y., Chee P., Isleib T.G., Holbrook C.C., Ozias-Akins P. (2019). Major seed size QTL on chromosome A05 of peanut (*Arachis hypogaea*) is conserved in the US mini core germplasm collection. Mol. Breed..

[31] Mondal S., Badigannavar A.M. (2019). Identification of major consensus QTLs for seed size and minor QTLs for pod traits in cultivated groundnut (*Arachis hypogaea* L.). 3 Biotech.

[32] Wang Z., Tao S., Liu S., Jia M., Cui D., Sun G., Deng Z., Wang F., Kong X., Fu M. (2022). A Multi-Omics Approach for Rapid Identification of Large Genomic Lesions at the Wheat Dense Spike (wds) Locus. Front. Plant Sci..

[33] Komura S., Jinno H., Sonoda T., Oono Y., Handa H., Takumi S., Yoshida K., Kobayashi F. (2022). Genome sequencing-based coverage analyses facilitate high-resolution detection of deletions linked to phenotypes of gamma-irradiated wheat mutants. BMC Genom..

[34] Liu G.-S., Li H.-L., Grierson D., Fu D.-Q. (2022). NAC Transcription Factor Family Regulation of Fruit Ripening and Quality: A Review. Cells.

[35] Singh S., Koyama H., Bhati K.K., Alok A. (2021). The biotechnological importance of the plant-specific NAC transcription factor family in crop improvement. J. Plant Res..

[36] Jeong J.S., Kim Y.S., Redillas M.C.F.R., Jang G., Jung H., Bang S.W., Choi Y.D., Ha S.-H., Reuzeau C., Kim J.-K. (2013). OsNAC5 overexpression enlarges root diameter in rice plants leading to enhanced drought tolerance and increased grain yield in the field. Plant Biotechnol. J..

[37] Mathew I.E., Das S., Mahto A., Agarwal P. (2016). Three Rice NAC Transcription Factors Heteromerize and Are Associated with Seed Size. Front. Plant Sci..

[38] Jiang D., Chen W., Dong J., Li J., Yang F., Wu Z., Zhou H., Wang W., Zhuang C. (2018). Overexpression of miR164b-resistant OsNAC2 improves plant architecture and grain yield in rice. J. Exp. Bot..

[39] Nelson D.R., Schuler M.A., Paquette S.M., Werck-Reichhart D., Bak S. (2004). Comparative Genomics of Rice and Arabidopsis. Analysis of 727 Cytochrome P450 Genes and Pseudogenes from a Monocot and a Dicot. Plant Physiol..

[40] Nelson D.R. (2006). Plant Cytochrome P450s from Moss to Poplar. Phytochem. Rev..

[41] Ito T., Meyerowitz E.M. (2000). Overexpression of a Gene Encoding a Cytochrome P450, CYP78A9, Induces Large and Seedless Fruit in Arabidopsis. Plant Cell.

[42] Adamski Nikolai M., Anastasiou E., Eriksson S., O’Neill Carmel M., Lenhard M. (2009). Local Maternal Control of Seed Size by KLUH/CYP78A5-dependent Growth Signaling. Proc. Natl. Acad. Sci. USA.

[43] Chakrabarti M., Zhang N., Sauvage C., Muños S., Blanca J., Cañizares J., Diez Maria J., Schneider R., Mazourek M., McClead J. (2013). A Cytochrome P450 Regulates a Domestication Trait in Cultivated Tomato. Proc. Natl. Acad. Sci. USA.

[44] van der Knaap E., Chakrabarti M., Chu Y.H., Clevenger J.P., Illa-Berenguer E., Huang Z., Keyhaninejad N., Mu Q., Sun L., Wang Y. (2014). What Lies Beyond the Eye: The Molecular Mechanisms Regulating Tomato Fruit Weight and Shape. Front. Plant Sci..

[45] Monforte A.J., Diaz A., Caño-Delgado A., van der Knaap E. (2014). The Genetic Basis of Fruit Morphology in Horticultural Crops: Lessons from Tomato and Melon. J. Exp. Bot..

[46] Wang Y., Zhang M., Du P., Liu H., Zhang Z., Xu J., Qin L., Huang B., Zheng Z., Dong W. (2022). Transcriptome Analysis of Pod Mutant Reveals Plant Hormones are Important Regulators in Controlling Pod Size in Peanut (*Arachis hypogaea* L.). PeerJ.

[47] Liu Y., Liu Q., Su H., Liu K., Xiao X., Li W., Sun Q., Birchler J.A., Han F. (2021). Genome-wide Mapping Reveals R-loops Associated with Centromeric Repeats in Maize. Genome Res..

[48] Li H., Durbin R. (2010). Fast and Accurate Long-read Alignment with Burrows–Wheeler transform. Bioinformatics.

[49] McKenna A., Hanna M., Banks E., Sivachenko A., Cibulskis K., Kernytsky A., Garimella K., Altshuler D., Gabriel S., Daly M. (2010). The Genome Analysis Toolkit: A MapReduce framework for analyzing next-generation DNA sequencing data. Genome Res..

[50] Liu Y., Wang C., Su H., Birchler J.A., Han F. (2021). Phosphorylation of Histone H3 by Haspin Regulates Chromosome Alignment and Segregation during Mitosis in Maize. J. Exp. Bot..

[51] Kim D., Langmead B., Salzberg S.L. (2015). HISAT: A Fast Spliced Aligner with Low Memory Requirements. Nat. Methods.

[52] Anders S., Pyl P.T., Huber W. (2015). HTSeq—A Python Framework to Work with High-throughput Sequencing Data. Bioinformatics.

[53] Love M.I., Huber W., Anders S. (2014). Moderated Estimation of Fold Change and Dispersion for RNA-seq Data with DESeq2. Genome Biol..

[54] Yu G., Wang L.-G., Han Y., He Q.-Y. (2012). clusterProfiler: An R Package for Comparing Biological Themes Among Gene Clusters. OMICS A J. Integr. Biol..

[55] Liu Y., Su H., Liu Y., Zhang J., Dong Q., Birchler J.A., Han F. (2017). Cohesion and Centromere Activity are Required for Phosphorylation of Histone H3 in Maize. Plant J..

