# Supplementary material for: Multi-Omics Profiling Identifies Candidate Genes Controlling Seed Size in Peanut
Source: Plants (Basel). 2022 Nov 28;11(23):3276. doi: 10.3390/plants11233276 (PMC9740956; doi:10.3390/plants11233276)
Supplement: Supplementary file 1 [file plants-11-03276-s001.zip › Supplemental figure.pdf]

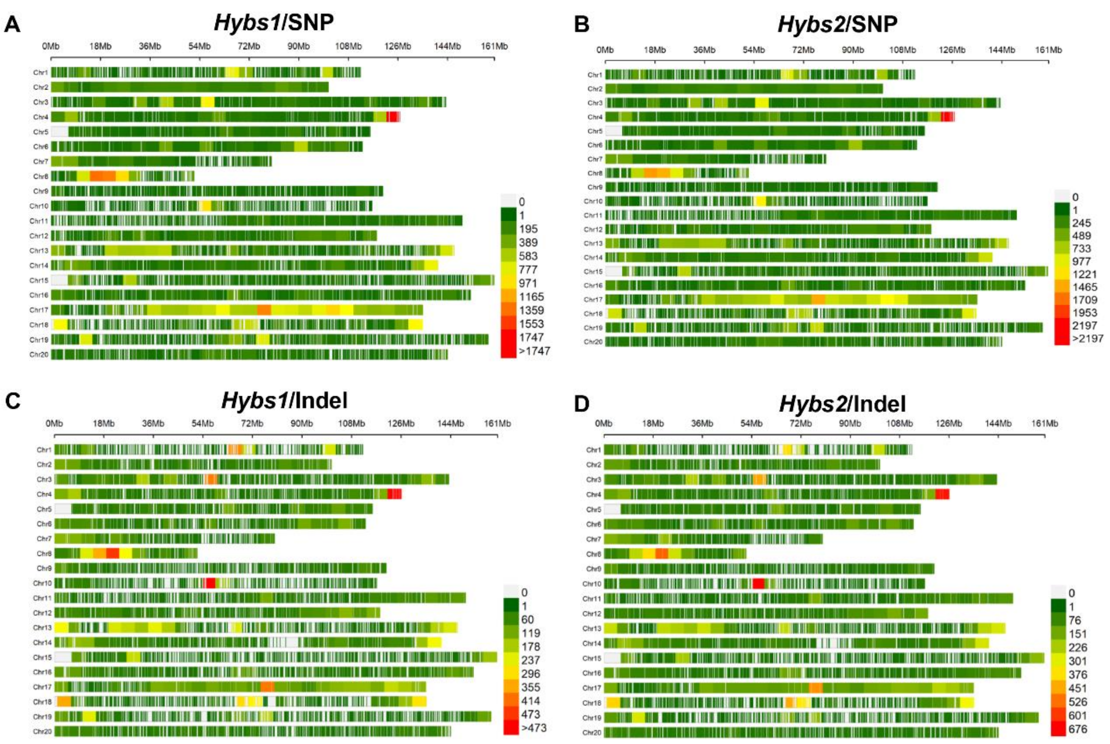

Figure S1. Manhattan plot for ^{60}Co-induced SNPs/indels.

(A) Manhattan plot for ^{60}Co-induced SNPs in *hybs1*.

(B) Manhattan plot for ^{60}Co-induced indels in *hybs1*.

(C) Manhattan plot for ^{60}Co-induced SNPs in *hybs2*.

(D) Manhattan plot for ^{60}Co-induced indels in *hybs2*.

The x axis showed the chromosomal position. Chr 1-10 indicated the A subgenome and chr 11-20 indicated the B subgenome.

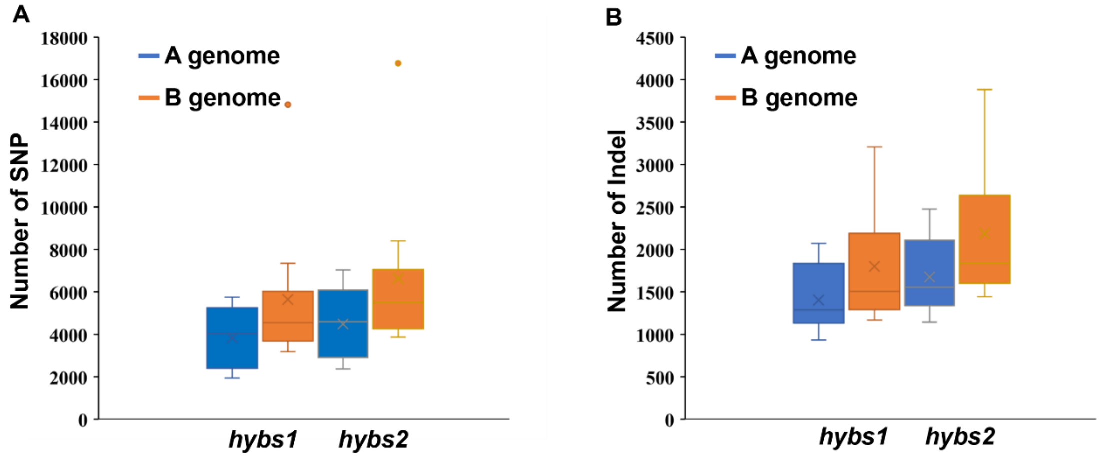

Figure S2. The number of ^{60}Co-induced SNPs/indels in *hybs1* and *hybs2*.

(A) Box-plots show the number of ^{60}Co-induced SNPs in different chromosome of *hybs1* and *hybs2*.

(B) Box-plots show the number of ^{60}Co-induced indels in different chromosome of *hybs1* and *hybs2*.

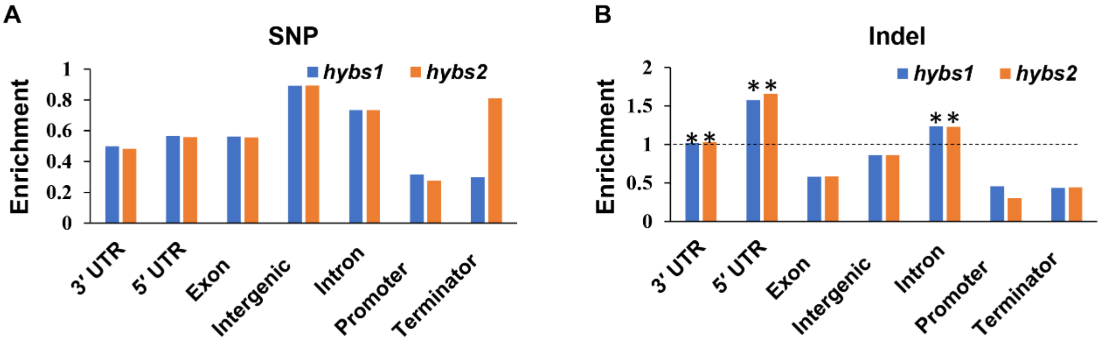

Figure S3. Relative enrichment of SNPs/Indels in different genomic elements.

(A) Relative enrichment of SNPs in different genomic elements.

(B) Relative enrichment of indels in different genomic elements.

The dashed line represents enrichment fold = 1.0. Permutation test; asterisks denote the observed value was >90% of the permutation value.

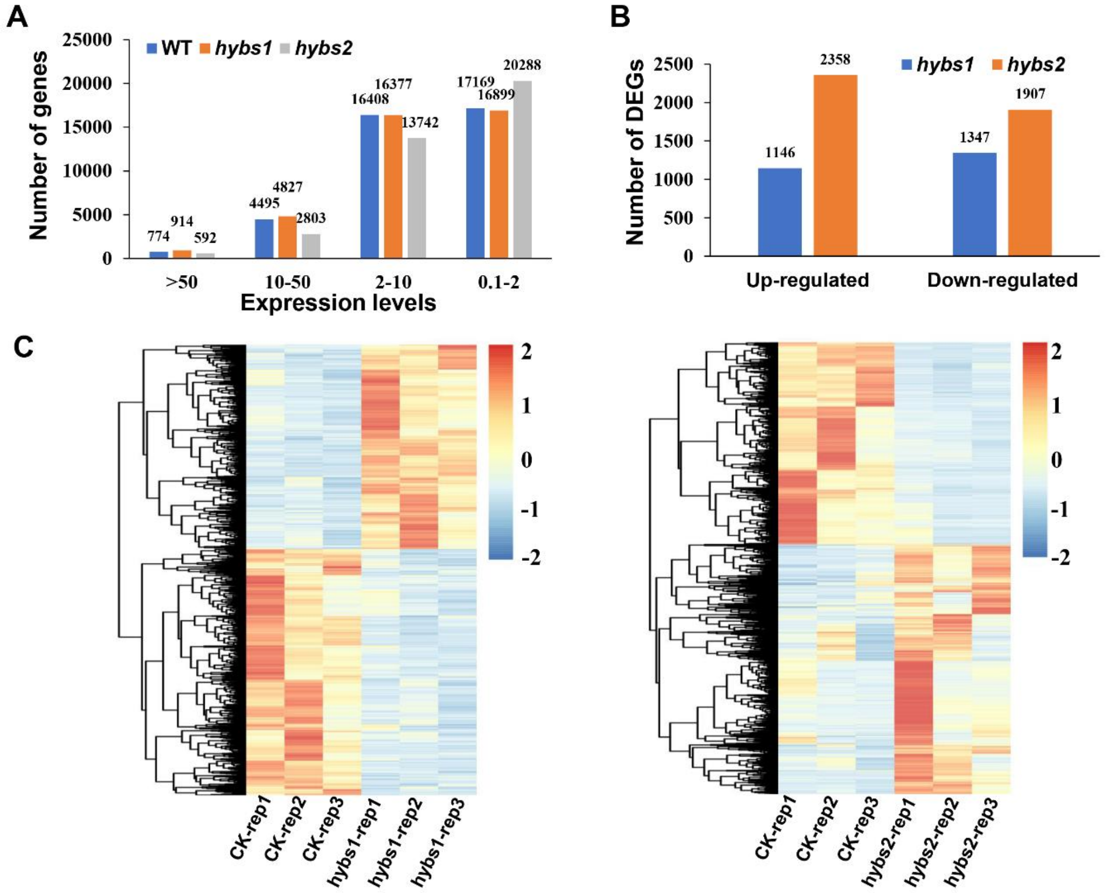

Figure S4. RNA-Seq analyses of *hybs1* and *hybs2*.

(A) The number of genes with different expression levels in WT, *hybs1*, and *hybs2*.

(B) The number of up-regulated and down-regulated genes in *hybs1* and *hybs2* as compared with WT.

(C) Heatmap showing the relative expression of genes in WT, *hybs1* and *hybs2*.

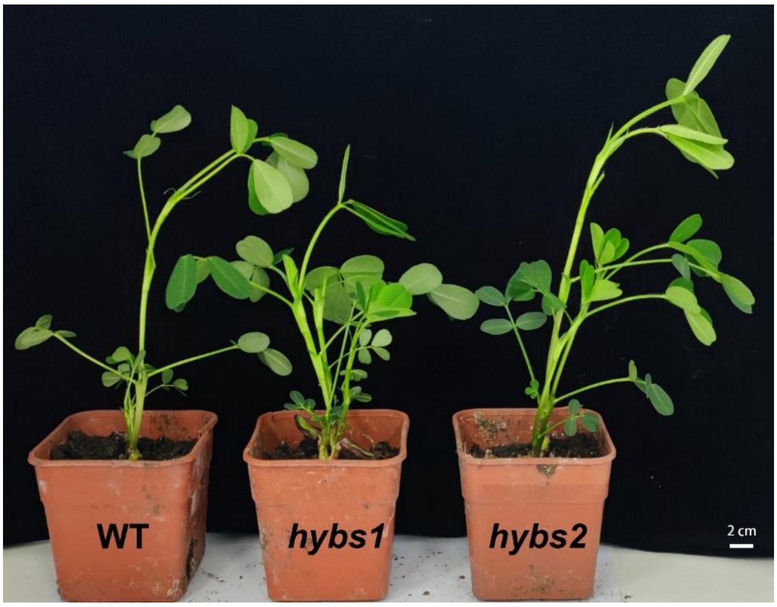

Figure S5. Plant height of WT, *hybs1*, and *hybs2*.

The phenotype of WT, *hybs1*, and *hybs2* in 10-d-old plants grown in a greenhouse under a 15-h light (26°C)/9-h dark (25°C) photoperiod. Scale bars are 2 cm.
