# Supplementary material for: Multi-Omics Profiling Identifies Candidate Genes Controlling Seed Size in Peanut
Source: Plants (Basel). 2022 Nov 28;11(23):3276. doi: 10.3390/plants11233276 (PMC9740956; doi:10.3390/plants11233276)
Supplement: Supplementary file 1 [file plants-11-03276-s001.zip › Supplemental Table.pdf]

Supplemental Table S1. Summary of sequencing data quality of WGS.

Samples	Clean read	Mappable read	Mappable rate	Unique mappable read	Unique mappable rate
**WT**	**313,413,740**	**159,426,571**	**50.87%**	**138,607,047**	**44.22%**
hybs1	**25,3469,704**	**129,604,577**	**51.13%**	**112,041,382**	**44.20%**
hybs2	**301,546,568**	**153,282,539**	**50.83%**	**132,721,777**	**44.01%**

Supplemental Table S2. Summary of sequencing data quality of RNA-seq.

Samples	Clean read	Mappable read	Mappable rate	Unique mappable read	Unique mappable rate
**WT-rep1**	**17,860,760**	**16,054,203**	**89.88%**	**13,623,941**	**76.28%**
**WT-rep2**	**16,183,301**	**14,406,000**	**89.02%**	**12,255,824**	**75.73%**
**WT-rep3**	**16,740,358**	**15,099,427**	**90.20%**	**9,863,762**	**58.92%**
***hybs1*-rep1**	**17,659,640**	**15,708,883**	**88.95%**	**13,564,218**	**76.81%**
***hybs1*-rep2**	**15,391,380**	**13,870,371**	**90.12%**	**11,873,311**	**77.14%**
***hybs1*-rep3**	**15,026,450**	**13,545,867**	**90.15%**	**9,763,765**	**64.98%**
***hybs2*-rep1**	**16,740,479**	**15,240,943**	**91.04%**	**12,594,265**	**75.23%**
***hybs2*-rep2**	**17,464,470**	**15,862,356**	**90.83%**	**8,165,127**	**46.75%**
***hybs2*-rep3**	**15,124,890**	**13,416,457**	**88.70%**	**7,519,683**	**49.72%**

Supplemental Table S3. List of oligos and primer sequences used in this study.

Primer	Sequence (5' to 3')	Assay
**TIF-134-3**	AAAAATATTTTTTTAGTTTAAAACTTAAAAAAATAAGAAATTTA	FISH
**TIF-165-3**	ATGCTTATGATAATTATGTGCGCATACTTTAATAGGTGCGATCAT	FISH
**TIF-185-1**	AAAAACCTAAGAAAACCATAGAAAATCCTA	FISH

**TIF-439**	AGGGTTTTTCTAGGGTTTTTCTAGGGTTTTTCT	FISH
**Ipa-1137**	TGCAACGGAGGCTGGGTGACGTTGCAACGGAGGCTGGGTGACGT	FISH
**Ipa-1162**	CCCAGCCTCCGTTGCAACGCGCCCAGCCTCCGTTGCAACGCG	FISH
**DP-1**	TGCGATCATACCAGCACTAATGCACCGGATCCCGTCAGAACTCTGAAGTTAAGCGTG	FISH
**DP-5**	TTTAGGGTTTAGGGTTTAGGGTTTAGGGTTTAGGGTTTAGGGTTTAGGGTTTAGGG	FISH
**J9EPM7-F1**	CCCCAATCCTTTGCACAACA	qPCR-RT
**J9EPM7-R1**	TTGTTCCCATTGGTGCCATG	qPCR-RT
**5UX7QI-F1**	GTGGTTTCAATGCCGCCTTA	qPCR-RT
**5UX7QI-R1**	CTCAACATGCCAGTGTCACC	qPCR-RT
**M4RITN-F1**	ATGGGAGCACGGGATATCTG	qPCR-RT
**M4RITN-R1**	GTAACCCGCTCACAACATCC	qPCR-RT
**FHX76J-F1**	TGCTGGAATTGCTGCTTTCA	qPCR-RT
**FHX76J-R1**	TCTTGCAGGCACCATTTTGA	qPCR-RT
**TMG43S-F1**	CCAAGAGTTACGAGGCATGC	qPCR-RT
**TMG43S-R1**	GCGAATGTCTTGTACGGCTT	qPCR-RT
**CDPA7L-F1**	TCTGTACTTCCCAATTCACTACC	qPCR-RT
**CDPA7L-R1**	CCCTTGAACTGTAGCTCCTTCT	qPCR-RT
**TMG43S-F2**	AATAAACCGTAACCGCACCC	Sanger
**TMG43S-R2**	ACTGTGCGAATGTCTTGTGC	Sanger
**CDPA7L-F2**	CCTTTACATGACATCAAACCTGG	Sanger
**CDPA7L-R2**	CCCCTTGAACTGTAGCTCCT	Sanger
